# Integrative transcriptomics-based identification of cryptic drivers of taxol-resistance genes in ovarian carcinoma cells: Analysis of the androgen receptor

**DOI:** 10.18632/oncotarget.4824

**Published:** 2015-08-11

**Authors:** Nian-Kang Sun, Shang-Lang Huang, Hsing-Pang Lu, Ting-Chang Chang, Chuck C.-K. Chao

**Affiliations:** ^1^ Department of Biochemistry and Molecular Biology, College of Medicine, Chang Gung University, Taoyuan, Taiwan, Republic of China; ^2^ Division of Biomedical Sciences, Chang Gung University of Science and Technology, Taoyuan, Taiwan, Republic of China; ^3^ Department of Obstetrics and Gynecology, Chang Gung Memorial Hospital Linkou Medical Center, Taoyuan, Taiwan, Republic of China; ^4^ Graduate Institute of Biomedical Sciences, College of Medicine, Chang Gung University, Taoyuan, Taiwan, Republic of China

**Keywords:** androgen receptor, taxol, ovarian cancer, transcription factor, multiple drug resistance

## Abstract

A systematic analysis of the genes involved in taxol resistance (txr) has never been performed. In the present study, we created txr ovarian carcinoma cell lines to identify the genes involved in chemoresistance. Transcriptome analysis revealed 1,194 overexpressed genes in txr cells. Among the upregulated genes, more than 12 cryptic transcription factors were identified using MetaCore analysis (including AR, C/EBPβ, ERα, HNF4α, c-Jun/AP-1, c-Myc, and SP-1). Notably, individual silencing of these transcription factors (except HNF4`)sensitized txr cells to taxol. The androgen receptor (AR) and its target genes were selected for further analysis. Silencing AR using RNA interference produced a 3-fold sensitization to taxol in txr cells, a response similar to that produced by silencing abcb1. AR silencing also downregulated the expression of prominent txr gene candidates (including abcb1, abcb6, abcg2, bmp5, fat3, fgfr2, h1f0, srcrb4d, and tmprss15). In contrast, AR activation using the agonist DHT upregulated expression of the target genes. Individually silencing seven out of nine (78%) AR-regulated txr genes sensitized txr cells to taxol. Inhibition of AKT and JNK cellular kinases using chemical inhibitors caused a dramatic suppression of AR expression. These results indicate that the AR represents a critical driver of gene expression involved in txr.

## INTRODUCTION

The taxanes paclitaxel (taxol) and docetaxel are microtubule-stabilizing agents that function primarily by interfering with spindle microtubule dynamics, ultimately causing cell cycle arrest and apoptosis. These agents have become widely recognized as active chemotherapeutic agents in the treatment of breast and ovarian cancer, among others. However, their therapeutic usefulness is limited by acquired de novo resistance [1, 2]. Membrane transporters from the ATP-binding cassette (ABC) and solute carrier (SLC) families play a major role in drug resistance. Probably the most important ABC protein in this context is glycoprotein P (P-gp) which is encoded by the ABCB1 gene (multidrug resistance protein 1, or MDR1) [3–5]. This protein functions as a drug efflux pump that can actively remove around 20 different cytostatic drugs from cancer cells. At least 10 additional ABC proteins may be involved in drug resistance [6]. Another group of membrane transporters involved in drug resistance is the SLC transporters, which function mainly as influx carriers [7]; these proteins are often downregulated in chemoresistant cells [8–10]. Spindle microtubules are the primary target of taxol. This chemotherapeutic drug disrupts microtubules by binding to their interior surface [11]. Specific checkpoint proteins such as BRCA1 and the spindle assembly checkpoint proteins MAD2, BUBR1, synuclein-gamma and aurora A have all emerged as potential biomarkers of taxol resistance. In addition, the effects of MDR-1/P-gp on taxol resistance were extensively studied *in vitro* but the data remain conflicting. Despite these advances, no valid biomarkers exist that can predict resistance to taxanes in breast cancer [1]. Overexpression of MDR-1/P-gp and altered expression of microtubule-associated proteins (MAPs), including tau, stathmin, and MAP4, may help identify patients at risk of recurrence and most likely to benefit from taxane treatment [2].

Androgen-dependent activation of the androgen receptor (AR) is required for prostate-specific antigen production and survival of both normal and malignant prostate epithelial cells. Androgen deprivation therapy via surgical or medical castration remains the standard form of treatment for clinically advanced prostate cancer. For a number of years, docetaxel was the only treatment showing proven survival benefit for patients with castration-resistant prostate cancers [12]. Steroid hormones have been involved in the development and progression of epithelial ovarian cancer, an observation which suggests a role for androgens in this context; for instance, chemotherapy has been shown to decrease androgen production by cancer cells [13]. Although the AR is expressed in both normal and cancerous ovaries, we possess a limited understanding of AR activity in ovarian cancer cells. Notably, a number of studies showed that AR is overexpressed in ovarian cancer [14–18]. Epidemiological evidence and laboratory data strongly support a critical role for androgens in the origin and promotion of epithelial ovarian cancer and have led to clinical trials designed to target the AR (reviewed in ref. [19]). There is evidence for cross-talk between androgen signaling and other signaling pathways. For example, AR activation by dihydrotestosterone (DHT) in SKOV3 ovarian cancer cells and ascites-derived OVCAS-16 cells prevented the growth inhibitory effect of transforming growth factor-beta (TGF-β), while DHT alone had no effect on cell growth [20]. The effect of DHT was associated with downregulation of TGF-β1 and TGF-β2 receptors. However, it is uncertain whether AR activity is an important target of taxol in treatment of ovarian cancer. Studies examining regulation of gene expression are needed to determine the mechanism underlying the activity of the AR in mediating cell response to chemotherapy.

Upon binding androgens, the AR mediates the effects of male sex steroids by binding to cis-elements in the regulatory regions of target genes and regulating expression of androgen-dependent genes.. AR is a transcription factor with eight exons encoding five functional domains: a large amino-terminal transactivation domain (encoded by exon 1) [21]; a central DNA-binding domain (encoded by exons 2 and 3); a carboxy-terminal, ligand-binding domain (encoded by exons 4–8); and a hinge region (between the DNA-binding domain and ligand-binding domain) that contributes to nuclear localization and protein degradation [22]. The DNA-binding domain of the AR recognizes a palindromic response element that comprises an inverted repeat of the 5′-AGAACA-3′ hexamer with a 3-nucleotide spacer, usually termed the canonical androgen/glucocorticoid response element (ARE/GRE) [23]. Comparison of AR-binding events in the epididymis and prostate of wild-type (wt) and SPARKI mice (whose AR DNA-binding domain has the second zinc finger replaced by that of the glucocorticoid receptor) revealed that AR achieves selective chromatin binding through a less stringent sequence requirement for the 3′-hexamer [24].

Considering that taxanes are important chemo-therapeutic agents for the treatment of various cancers, we examined the mechanism of resistance in order to optimize the use of these drugs during cancer treatment. Using a microarray analysis, we searched for new candidate genes that may play a role in taxol resistance (txr). Using MetaCore analysis, we predicted driver genes involved in txr. Functional analysis of the identified driver genes and downstream target genes using RNA interference (RNAi) significantly sensitized txr cells to taxol compared to parental cells. Specifically, the AR was selected to further study the mechanism of activation of target txr genes in the context of taxol resistance. Our results provide several insights to build the gene network involved in txr.

## MATERIALS AND METHODS

### Cell lines and reagents

SKOV3 cells (American Type Culture Collection, Rockville, MD, USA) were grown as monolayers in a 1:1 mixture of DMEM/nutrient F-12 Ham (Life Technologies, Grand Island, NY, USA) supplemented with 1% (w/v) penicillin/streptomycin and 10% (v/v) fetal bovine serum (FBS) at 37°C in a humidified atmosphere containing 5% CO_2_. The chemotherapeutic drugs used included vincristine, taxol (paclitaxel), and cisplatin (Bristol-Myers Squibb, New York, NY, USA). Unless indicated otherwise, chemicals were purchased from Sigma-Aldrich (St. Louis, MO, USA). All reagents were used according to the instructions provided by the supplier.

### Taxol-resistant cell lines

Taxol-resistant ovarian cancer cells were prepared from the parental, drug-sensitive, ovarian cancer cell line SKOV3 by administrating taxol in a conventional dose-escalation manner. The concentration of taxol was increased stepwise, starting at 50 nM and ending at 600 nM. Parental SKOV3 cells were first exposed to 50 nM of taxol for 2 months followed by exposure to stepwise double concentrations of taxol for a further 2 months of treatment. Chemo-resistant cell lines were maintained in selective medium containing the taxol concentration used for selection of resistance. The cells were cultured in taxol-free medium for one week before further studies. Periodic evaluation of half-maximal inhibitory concentrations (IC_50_) confirmed that the txr phenotype was stable for at least 2 months in a drug-free medium.

### Cell viability assay

Cell viability was determined using the *in vitro* MTT [3-(4, 5-dimethylthiazol-2-yl)-2, 5-diphenyl-2*H*-tetrazolium bromide] colorimetric method as previously described [25]. One hundred μl of cells was seeded at a density of 3 × 10^4^ cells/ml in 96-well microplates. Cells were exposed to taxol in culture medium at 37°C for 72 h. Twenty μl of MTT solution (5 mg/ml in PBS) was added to each well, prior to incubation for 4 h. Optical density (OD) of the purple formazan product was measured at a wavelength of 540 nm using a spectrophotometer. The 50% inhibitory concentrations (IC_50_) of cell proliferation or cell viability were defined as the levels that respectively cause 50% reduction in cell viability versus the DMSO-treated control.

### Quantitative PCR analysis

Total RNA was extracted with the Trizol reagent (Life Technologies) as previously described [26]. RNA concentrations were assessed using a spectrophotometer, and only the samples with an A_260_/A_280_ ratio between 1.9 and 2.2 were used. Real-time quantitative PCR was performed on total RNA as before [26].

### Oligonucleotide DNA microarray

Fluorescent RNA targets were prepared from 5 μg total RNA of SKOV3 and taxol-resistant derivative cells using OneArray Amino Allyl aRNA Amplification Kit (Phalanx Biotech Group, Hsinchu, Taiwan) and Cy5 dyes (GE Healthcare, Little Chalfont, UK). Fluorescent targets were hybridized to the Human Whole Genome OneArray v6.1 microarray, which contains 31, 741 DNA oligonucleotide probes (HOA6.1; Phalanx Biotech Group) with Phalanx OneArray Plus Hybridization Protocol. The slides were dried by centrifugation, followed by scanning using the Agilent G2505C Microarray Scanner and GenePix software GenePix Pro 4.1.1.44 (Molecular Devices, Sunnyvale, CA, USA) to obtain background subtracted and spatially de-correlated processed signal intensities. The signal intensity of each spot was transferred to the Rosetta Resolver System (Rosetta Biosoftware, Seattle, WA, USA) for data analysis. The error model of the Rosetta Resolver System removed both systematic and random errors. Spots that passed the selection criteria were normalized using the median scaling normalization method. The original DNA microarray data was deposited into the GEO database (GSE58840). Normalized spot intensities were transformed to gene expression log 2 ratios between parental and resistant groups under Rosetta Resolver error model adjustment. Fold change values were calculated from adjusted log 2 ratios and were used for selecting differentially-expressed genes. Independent *t*-tests were used to evaluate statistical significance. Genes whose expression levels were higher than the assumed threshold (upregulated > 2 fold and downregulated < 2 fold) were identified using the scatter plot method and selected for further analysis.

### Gene network analysis

Genes found to be overexpressed in taxol-resistant SKOV3/Tx600 cells were extracted and subsequently used to build a network in the software MetaCore Analytical Suite 6.13 build 61585 (Thomson Reuters, Philadelphia, PA, USA). The 1, 194 upregulated genes of the DNA microarray analysis dataset (GSE58840) that were expressed more than 2 fold in SKOV3/Tx 600 cells compared to parental SKOV3 cells were considered. Using the “transcription regulation” algorithm, we generated a transcription regulation network, and used the “direct interactions” algorithm between network objects (txr genes) with top ranked transcription drivers in a default, high trust *p* value set. AR was selected as an interesting element for building the network. The 112 genes that were expressed more than 10-fold in SKOV3/Tx 600 cells compared to parental SKOV3 cells are partially listed in Table 1. Among the algorithms available, the “direct interactions” function was selected to build the network, and no other element from the MetaCore database was added. For easy visualization, some unlinked genes were omitted in the network. Nine transcription factors including the AR were selected for further experiments.

**Table 1:** Upregulated genes in taxol resistant SKOV3/Tx600 cells and associated transactivators

Symbol NCBI (NM_ID)	Function	Ratio, SKOV3/Tx600 / SKOV3	
		Microarray	qPCR
***AP-1***			
CCL2 (NM_002982)	Chemokine	100.00	737.66 ± 34.01
ABCB1 (NM_000927)	Transporter	96.98	743.94 ± 42.14
TLR4 (NM_003266)	Single transmembrane cell-surface receptors	62.58	124.21 ± 9.71
PPARGC1 (NM_013261)	Transcriptional coactivator	30.92	88.64 ± 5.13
BMP4 (NM_001202)	Induction of cartilage and bone formation	27.42	100.30 ± 14.87
SNCA (NM_000345)	Regulation of dopamine release and transport	20.69	15.12 ± 0.34
INHBE (NM_031479)	Inhibition of follitropin secretion	19.04	8.41 ± 1.12
SPP1 (NM_000582)	Cytokine	15.31	8.91 ± 2.20
LCN2 (NM_005564)	Iron-trafficking protein	11.49	8.14 ± 1.12
MYLK1 (NM_053025)	Calcium/calmodulin-dependent myosin light chain kinase	10.43	20.14 ± 1.98
***AR***			
ABCB1 (NM_000927)	Transporter	96.98	743.94 ± 42.14
PEG10 (NM_001040152)	Prevention of apoptosis in hepatocellular carcinoma (HCC) cells	41.47	24.61 ± 1.44
H1F0 (NM_005318)	Nucleosome structure	31.00	63.17 ± 8.97
TMPRSS15 (NM_002772)	Scavenger receptor and serine-type endopeptidase	30.63	8.75 ± 1.21
FAT3 (NM_001008781)	Calcium ion binding	18.70	9.78 ± 1.29
SPP1 (NM_000582)	Cytokine	15.31	8.91 ± 2.20
BMP5 (NM_021073)	Cytokine activity	15.30	5.58 ± 0.86
SRCRB4D (NM_080744)	Scavenger receptor activity	14.70	3.03 ± 0.12
FGFR2 (NM_022970)	Cell-surface receptor	13.50	6.19 ± 0.94
LCN2 (NM_005564)	Iron-trafficking protein	11.49	8.14 ± 1.12
PSCA (NM_005672)	Glycosylphosphatidylinositol-anchored cell membrane glycoprotein	10.08	13.66 ± 2.13
ABCB6 (NM_005689)	Transporter	8.09	5.90 ± 0.64
ABCG2 (NM_004827)	Channel	4.70	17.96 ± 0.49
***C/EBPβ***			
CCL2 (NM_002982)	Chemokine	100.00	737.66 ± 34.01
ABCB1 (NM_000927)	Transporter	96.98	743.94 ± 42.14
PPARGC1 (NM_013261)	Transcriptional coactivator	30.92	88.64 ± 5.13
INHBE (NM_031479)	Inhibition of follitropin secretion	19.04	8.41 ± 1.12
SPP1 (NM_000582)	Cytokine	15.31	8.91 ± 2.20
CD34 (NM_001025109)	Adhesion molecule	13.97	11.42 ± 2.14
LCN2 (NM_005564)	Iron-trafficking protein	11.49	8.14 ± 1.12
***ERα***			
ABCB1 (NM_000927)	Transporter	96.98	743.94 ± 42.14
INHBE (NM_031479)	Inhibition of follitropin secretion	19.04	8.41 ± 1.12
SPP1 (NM_000582)	Cytokine	15.31	8.91 ± 2.20
LCN2 (NM_005564)	Iron-trafficking protein	11.49	8.14 ± 1.12
***HNF4a***			
CYB5A (NM_001190807)	Membrane-bound electron carrier cytochrome	39.72	29.27 ± 1.99
INHBE (NM_031479)	Inhibit of follitropin secretion	19.04	8.41 ± 1.12
BMP5 (NM_021073)	Cytokine activity	17.35	249.74 ± 16.89
RPS19 (NM_001022)	Cytoplasmic ribosomal protein	16.96	37.94 ± 2.11
SPP1 (NM_000582)	Cytokine	15.31	8.91 ± 2.20
LCN2 (NM_005564)	Iron-trafficking protein	11.49	8.14 ± 1.12
MYLK1 (NM_053025)	Calcium/calmodulin-dependent myosin light chain kinase	10.43	20.14 ± 1.98
***C-Myc***			
BEX2 (NM_001168399)	Regulator of mitochondrial apoptosis and G1 cell cycle	58.24	27.72 ± 2.93
FGFR2 (NM_022970)	Cell-surface receptor	57.17	169.11 ± 11.01
PEG10 (NM_001040152)	Prevention of apoptosis in hepatocellular carcinoma (HCC) cells	41.47	24.61 ± 1.44
CDH5 (NM_001795)	Calcium-dependent cell-cell adhesion glycoprotein	41.35	43.58 ± 4.34
CYB5A (NM_001190807)	Membrane-bound electron carrier cytochrome	39.72	29.27 ± 1.99
CCNA1 (NM_001111045)	Regulators of CDK kinases	34.60	79.15 ± 4.33
H1F0 (NM_005318)	Nucleosome structure	30.97	256.88 ± 13.41
RPL3 (NM_000967)	Cytoplasmic ribosomal protein	28.79	19.88 ± 2.01
BMP4 (NM_001202)	Induction of cartilage and bone formation	27.42	100.30 ± 14.87
RPS19 (NM_001022)	Cytoplasmic ribosomal protein	16.96	37.94 ± 2.11
SPP1 (NM_000582)	Cytokine	15.31	8.91 ± 2.20
LCN2 (NM_005564)	Iron-trafficking protein	11.49	8.14 ± 1.12
NPDC1 (NM_015392)	Suppression of oncogenic transformation	11.41	41.23 ± 2.16
***SP1***			
ABCB1 (NM_000927)	Transporter	96.98	743.94 ± 42.14
TLR4 (NM_003266)	Single transmembrane cell-surface receptors	62.58	124.21 ± 9.71
CDH5 (NM_001795)	Calcium-dependent cell-cell adhesion glycoprotein	41.35	43.58 ± 4.34
H1F0 (NM_005318)	Nucleosome structure	30.97	256.88 ± 13.41
PPARGC1 (NM_013261)	Transcriptional coactivator	30.92	88.64 ± 5.13
RPL3 (NM_000967)	Cytoplasmic ribosomal protein	28.79	19.88 ± 2.01
BMP4 (NM_001202)	Induction of cartilage and bone formation	27.42	100.30 ± 14.87
SNCA (NM_000345)	Regulation of dopamine release and transport	20.69	15.12 ± 0.34
INHBE (NM_031479)	Inhibition of follitropin secretion	19.04	8.41 ± 1.12
RPS19 (NM_001022)	Cytoplasmic ribosomal protein	16.96	37.94 ± 2.11
SPP1 (NM_000582)	Cytokine	15.31	8.91 ± 2.20
CD34 (NM_001025109)	Adhesion molecule	13.97	11.42 ± 2.14
NPDC1 (NM_015392)	Suppression of oncogenic transformation	11.41	41.23 ± 2.16

Highly upregulated genes identified by DNA microarray and q-PCR in taxol-resistant SKOV3/Tx600 cells. These genes are arranged by grouping under predicted transcription factors. Their NCBI ID and major functions are also indicated. The original DNA microarray data was deposited into the GEO database (GSE58840). The q-PCR experiments were performed in triplicate.

### Western blotting

Cells were washed twice with ice-cold phosphate-buffered saline (pH 7.4) and lyzed on ice for 30 min using standard cell lysis buffer. After centrifugation for 15 min at 4°C, the supernatant was removed and protein concentration was determined using the Bio-Rad protein assay (Bio-Rad, Hercules, CA, USA). Fifty μg of proteins from each sample was separated on a 10% sodium dodecyl sulfate-polyacrylamide gel and electro-blotted onto polyvinylidene difluoride (PVDF) membranes (Millipore, Bedford, MA, USA). After electroblotting, the membranes were blocked in 5% non-fat dry milk (dissolved in 0.1 M Trizma base, 0.15 M NaCl, 0.05% Tween 20, pH 7.4). Membranes were incubated with primary antibodies raised against either MDR1 or GAPDH (Santa Cruz Biotechnology, Santa Cruz, CA, USA) in the blocking solution containing 3% non-fat dry milk.

### Knockdown assay

Knockdown of candidate genes was performed using pLKO.1 plasmids expressing shRNA purchased from the National RNAi Core Facility (Academia Sinica, Taipei, Taiwan) as described before [27]. Luciferase shRNA (TRCN0000072244) was used as a negative control. Specific shRNA knockdown clones were selected for cell viability assay using puromycin. shRNA plasmids encoding genes highly overexpressed in taxol-resistant cells were selected and used in the present study. Both shRNA clone ID and target sequence were included: BMP5 (TRCN0000371431, ATGCCACCAACCACGCTATAG), FGFR2 (TRCN0000219680, TGGAGTACTCCTATGACATTA), ABCB1 (TRCN0000059684, GCAGCAATTA GAACTGTGATT), ABCG2 (TRCN0000059802, CCTGCCAATTTCAAATGTAAT), ABCB6 (TRCN000 0060320, GAACCAAGTTTCGTCGTGCTA), FAT3 (TRCN0000338931, TAATAGACAGGGACCATATTT), H1F0 (TRCN0000106875, GCCCTGTTGAAACTTA GGTTT), SRCRB4D (TRCN0000056807, CCTCCTA CGACACTGCCGAAT), TMPRSS15 (TRCN0000195271, CTGATGCTCTAACGTGTATAA), AR (TRCN00 00003715, CCTGCTAATCAAGTCACACAT), Myc (TRCN0000039638, CCATAATGTAAACTGCCTCAA), Jun (TRCN0000338221, GCTAACGCAGCAGTTGC AAAC), STAT3 (TRCN0000329886, GCAAAGAATC ACATGCCACTT), PPARγ (TRCN0000001673, CAGCATTTCTACTCCACATTA), CEBPβ(TRCN 0000007441, CCTGCCTTTAAATCCATGGAA), ERα (TRCN0000338158, GTGTGCCTCAAATCTATTATT), SP1 (TRCN0000274153, GGCAGATCTGCAGTCC ATTAA), HNF4α (TRCN0000376470, TCACCTGATGC AGGAACATAT).

## RESULTS

### Transcriptomic analysis of taxol-resistant cells and identification of txr driver genes

**Figure 1:**
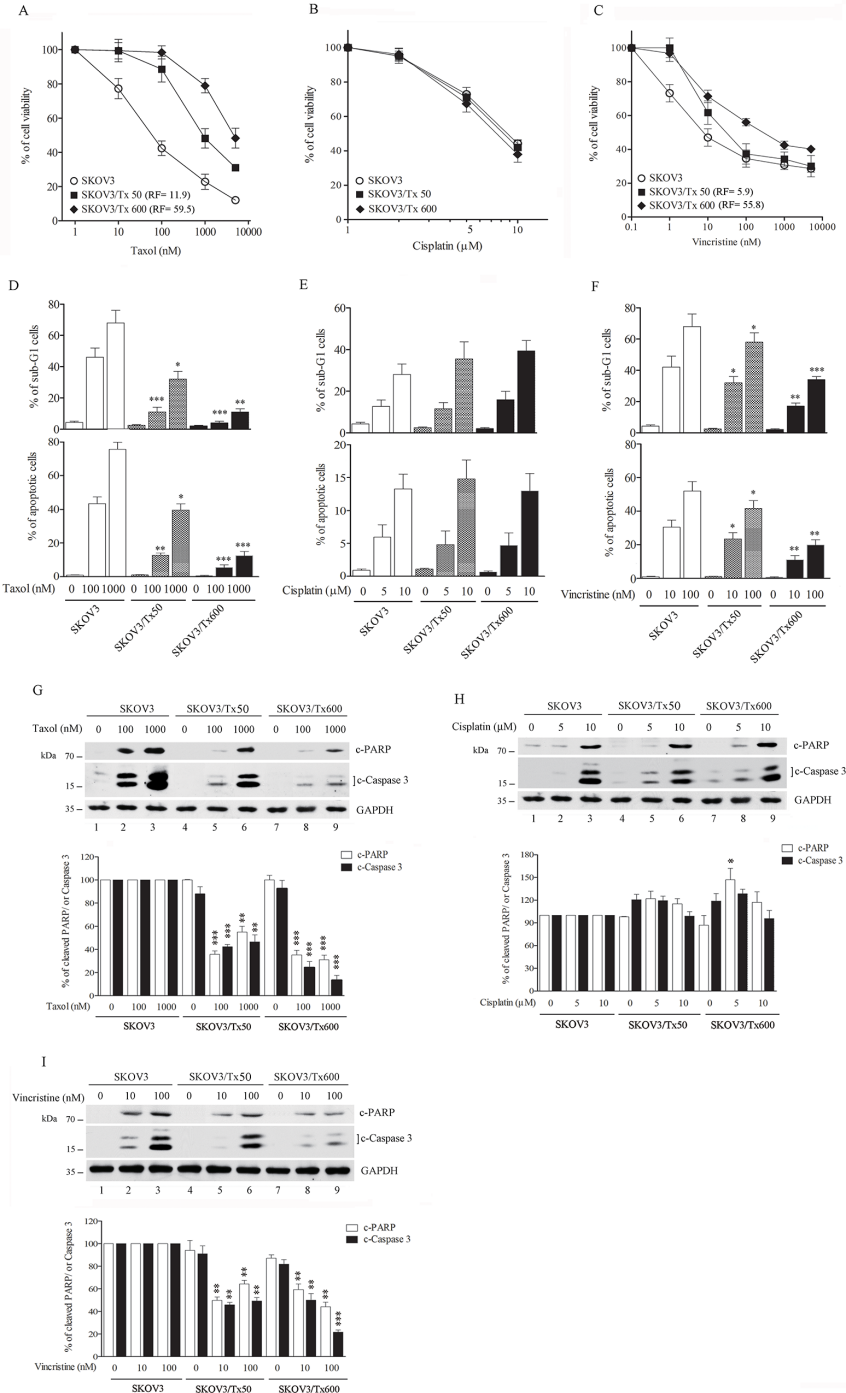
Establishment of txr cells **A–C.** Cell viability in response to taxol, cisplatin, and vincristine. **D–F.** Apoptotic sub-G1 cells in response to chemotherapeutic drugs. **G–I.** Caspase activation in response to taxol, cisplatin, and vincristine. The experiments were performed in triplicate (*p < 0.05, **P < 0.01, ***P < 0.005).

To access the mechanism of taxol resistance, we prepared txr cells by treating SKOV3 cells with increasing concentrations of taxol (see Materials and Methods). Cell viability to various chemotherapeutic drugs was then examined. The txr cell lines SKOV3/Tx50 and SKOV3/Tx600 displayed resistance to both taxol (Fig. 1A) and vincristine (Fig. 1C). However, the cell lines showed no resistance to cisplatin (Fig. 1B). A resistance factor (RF), calculated as the IC_50_ of txr cells divided by the IC_50_ of parental cells, was used to quantify the level of resistance (Fig. 1A and 1C, RFs are given within parentheses). Monitoring of sub-G1 cells revealed that txr cells showed significantly reduced apoptosis levels in response to both taxol and vincristine, whereas response to cisplatin was similar in parental and txr cells (Fig. 1D–1F). Similarly, caspase activation analysis indicated that txr cells showed reduced caspase activation and c-PARP cleavage following treatment with taxol and vincristine, while no significant change was noted for cisplatin (Fig. 1G–1I). Taken together, these results indicate that SKOV3/Tx50 and SKOV3/Tx600 cells are moderately and highly resistant to taxol, respectively.

To study the molecular basis of txr, we used a whole genome microarray to compare the transcriptome profile of parental and highly-resistant SKOV3/Tx600 cells. We identified 2, 677 genes that were differentially expressed at least 2 fold between these cells, including 1, 194 upregulated genes and 1, 483 downregulated genes. Using q-PCR, we confirmed that the most upregulated genes in the microarray data were indeed overexpressed (Table 1; also including ABCG2 and ABCB6 which were less upregulated compared to other genes). The primer sequences used for each gene are shown in Table 2. Three patterns of upregulated genes were noticed [28]: 1) similarly upregulated in both poorly and highly resistant cells; 2) upregulated 2–4 fold in poorly resistant cells and over 4 fold in highly resistant cells; and 3) upregulated over 4 fold in poorly resistant cells and 2–4 folds in highly resistant cells (data not shown). For example, in the first category, 716 and 684 genes were upregulated in poorly and highly resistant cells, respectively, while 94 genes were upregulated in both cells. Notably, these common upregulated genes were not enriched in specific metabolic pathways. For instance, the top-ranking pathway in ATM/ATR regulation of cell cycle checkpoint covered only two genes (gadd45a and cds1) (*P* < 0.005). In the third category, 196 and 1, 017 genes were upregulated in poorly and highly resistant phenotypes, respectively, while 47 genes were upregulated in both types of cells. Genes of the third category, which were highly upregulated in the early stage (poorly resistant cells) following exposure of parental cells to taxol but which showed a low level of upregulation in the later stage (highly resistant cells), are potentially required for the upregulation of resistance genes. The “early onset” genes such as FKBP5/AR [28] and those involved in the development of txr appear to be worthy of further studies.

**Table 2:** Primers for q-PCR analysis of genes associated with txr phenotype

Genes	Forward	Reverse
***txr genes***		
ABCB1	GTTCAAACTTCTGCTCCTGA	CCCATCATTGCAATAGCAGG
ABCB6	GAGCAGGGCCCCTTCGCTTT	AGTCTCCCGCCCATCGGCAT
ABCG2	GGCACTGGCCATAGCAGCAG	AGCCATGACAGCCAAGATGCAA
BEX2	AGTTTGCGGGCAGTCAGCACT	GGGAAACCATCAGGATTCAGGGCA
BMP4	GGGCCATGCCTTGACCCGAC	GAGTGGCGCCGGCAGTTCTT
BMP5	GGCAGAAGAGACCAGAGGGGCA	TGGGTGGTCAGAGGAGTCGTCC
CCL2	GAAGCTCGCACTCTCGCCTCC	TGAGCGAGCCCTTGGGGAATGA
CCNA1	CAGATTTCGTCTTCCAGCAGCAG	CGGGGCTCTGGTGAGTATC
CD34	AGGAGAAAGGCTGGGCGAAG	GAATGGCCGTTTCTGGAGGT
CDH5	ATGCGGCTAGGCATAGCATT	TGTGACTCGGAAGAACTGGC
CYB5A	ACAAGCCTCCGGAAACTCTTA	GGAGGTGTTCAGTCCTCTGC
FAT3	CGGCCGCAACGTCTACCAGG	TCAGGATGCGGGGCGACTCA
FGFR2	GAGTTGCTCCCCGCAACCCC	CCGCGACCTGTGTTGTCCCC
H1F0	TGGCTGCCACGCCCAAGAAA	TCTTGCCGGCCCTCTTGGCA
INHBE	GTCAAGACGGATGTGCCAGA	ATGCCTCCAGTCACAGATGC
LCN2	ACCCTCTACGGGAGAACCAA	CAGGGAGGCCCAGAGATTTG
MYLK1	TACCTCTGCCTGCTGAAAGC	CCTTTCCACTTGGAGGGTCC
NPDC1	ATGCTGTGTGCTTTTGGCTG	GGGGGTCTAAACCGAACAGG
Peg10	TCCACGAAACTCACGACCTG	CCCTAGGACGACAGGGAAGA
PPARGC1	TGCATGAGTGTGTGCTCTGT	GCACACTCGATGTCACTCCA
PSCA	CTGAGGCACATCCTAACGCA	TCAATAGAGCCGATCTGCCG
RBP7	CACGAACAGCAGCCTAAGGA	GGGTCCAGCCTCTGTTCTTC
RPL3	CATCCGTGTCATTGCCCACA	TGCCCAAACACTTGGTTCAC
RPS19	AGTCCCCGAATGGGTGGATA	TCTTGGTCATGGAGCCAACC
SNCA	CAGGAACAGCTGTCTTCCAGCTC	GCTGCTTCTGCCACACCCTGT
SPP1	AGGCTGATTCTGGAAGTTCTGAG	TTACTTGGAAGGGTCTGTGGGG
SRCRB4D	TGGGGGTGGAGGTTGGGAGATG	TGGCCAGTGGCAGGAGGAGAA
TLR4	GGATCAAGGACCAGAGGCAG	AGGCATGCCCTGCTTATCTG
TMPRSS15	TATGGCGGCCGACTGCTCTG	TACACGCAGTGTGCGGCGG
***TFs***		
AR	CGGAAGCTGAAGAAACTTGG	ATGGGCTGACATTCATAGCC
C/EBPβ	GACAAGCACAGCGACGAGTA	AGCTGCTCCACCTTCTTCTG
ERα	TGTACCTGGACAGCAGCAAG	CTCGGAGACACGCTGTTGAG
HNF4α	TCAAGAAATGCTTCCGGGCT	GGCTGCTGTCCTCATAGCTT
Jun	GAGCTGGAGCGCCTGATAAT	CCCTCCTGCTCATCTGTCAC
Myc	TCAAGAGGCGAACACACAAC	GGCCTTTTCATTGTTTTCCA
SP1	TCTTCCTCCTCTGGGGCTAC	CCACCAGAGACTGTGCGATT

**Figure 2:**
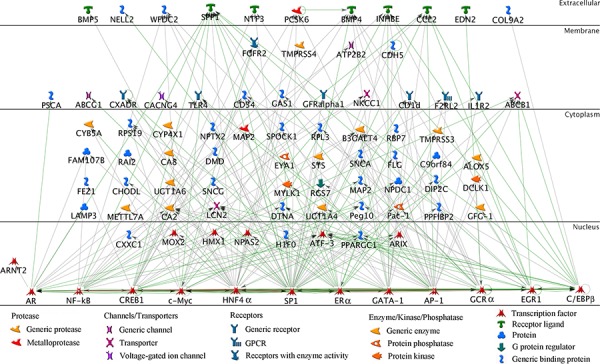
Network of txr genes MetaCore analysis of transcriptome profiling identifies drivers for upregulated txr genes. 112 genes that were overexpressed over 10-fold in SVKO3/Tx600 cells were subjected to MetaCore analysis by setting AR as target gene (see Materials and Methods). Note that AR and other prominent gene products/transcription factors or drivers were identified (NFκB, CREB1, c-Myc, HNF4α, SP1, ERα, CATA1, c-Jun/AP1, GCRα, EGR1, and C/EBPβ). Each driver gene product interacts with unique target txr genes. Only direct interactors are shown. Green lines, positive regulations according to public database; gray lines, unspecified but potentially novel interactions. Symbols are indicated at bottom.

The upregulated genes identified in txr cells were analyzed using the MetaCore platform (see Materials and Methods) in order to reveal potential connections between gene activities involved in resistance. A network analysis of these genes suggested induction of transcriptional factors that are too low to be detected by microarray. We did not identify an enriched cluster of upregulated genes that are involved in specific cell functions by this method. Twelve genes that interact with a large number of genes were indicated (including AP1/c-Jun, AR, C/EBP, ERα, HNF4-α, c-Myc, and SP1). These driver genes encode nuclear transcription factors and their interacting gene products are indicated in Fig. 2. These transcription factor genes showed more than ten interactions each. In addition, they, AR and c-Myc for example, appeared to mutually regulate each other. Genes that were upregulated more than ten fold in SKOV3/Tx600 cells were grouped as specific driver genes according to Metacore analysis, and their expression levels were confirmed by qPCR analysis. The genes identified are briefly described in Table 1.

### Silencing “cryptic” or minimally-upregulated driver genes causes taxol sensitization

**Figure 3:**
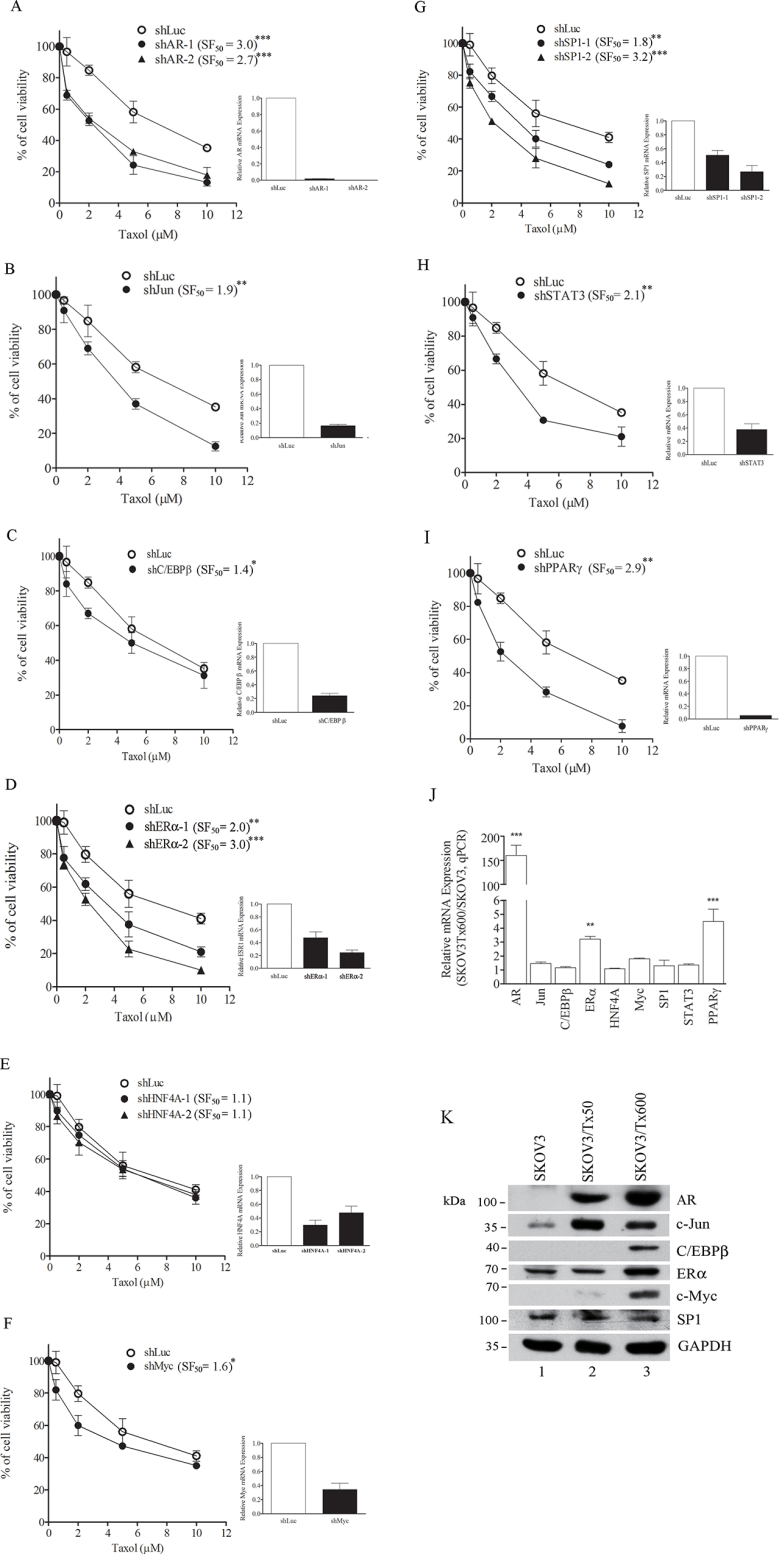
Silencing of driver genes sensitizes taxol response in txr cells **A–I.** Modulation of cell viability following silencing of AR, Jun, C/EBPβ, ERα, HNF4α, c-Myc, SP1, STAT3, and PPARγ. The silencing efficiency and sensitization factor (SF) for each gene are indicated. **J.** Minimal upregulation of driver genes. Relative mRNA determined by q-PCR was calculated based on three independent experiments. Only c-Myc and STAT3 produced statistically significant results (P < 0.05). **K.** Western blotting of AR, c-Jun, C/EBPβ, ERα, c-Myc, and SP1 in txr cells.

To test the possibility that the identified driver txr genes regulate cell sensitivity to taxol, we silenced these genes individually. Silencing of AR sensitized SKOV3/Tx600 cells to the drug (SF_50_=3.0; Fig. 3A). The sensitization factor (SF_50_) was defined as the concentration that reduces cell viability by 50% (IC_50_) in the gene silencing treatment divided by the IC_50_ of shLuc-treated control. Silencing the other driver genes also sensitized txr cells to taxol: c-Jun (SF_50_=1.9), C/EBPβ (SF_50_=1.4), ERα (SF_50_=3.0), c-Myc (SF_50_=1.6), SP1 (SF_50_=3.2), STAT3 (SF_50_=2.1), and PPARγ (SF_50_=2.9; Fig. 3B–3I). However, silencing HNF4α did not produce a significant level of sensitization (SF_50_=1.1; Fig. 3E). It should be noted that the driver genes were considered “cryptic” drivers because they were only minimally overexpressed compared to parental cells, and some did not reach a statistical significant level of upregulation (Fig. 3J). However, their upregulated protein levels were readily detected in txr cells, except for SP1 (Fig. 3K, six proteins are shown for examples). Interestingly, only minimal or no sensitization to cisplatin was detected following silencing of the driver genes in parental cells (data not shown). Furthermore, silencing the driver genes sensitized other ovarian carcinoma cell lines to taxol; for instance, knockdown of the AR sensitized MDAH-2774 and TOV21G cell lines to taxol at levels similar to those observed for SKOV3/Tx600 cells (SF_50_ = 2.2 and 2.9, respectively; data not shown). Notably, chromatin immunoprecipitation analysis (ChIP) indicated that AR was constitutively bound to six txr genes (selected at random), and that four of these genes were upregulated following activation of AR (Figure S1). These results suggest that the driver gene products at protein level and/or transactivation activity may play an important role in txr by upregulating their target genes.

### AR expression and nuclear location affect taxol sensitivity

**Figure 4:**
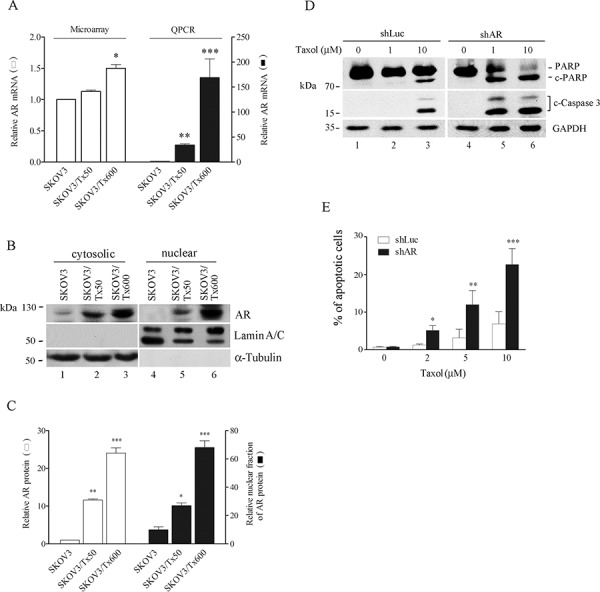
AR expression and nuclear location is associated with taxol sensitivity **A.** Enhanced AR mRNA expression in txr cells. **B–C.** Enhanced nuclear AR levels in txr cells. Representative Western blotting is shown in (B) and quantitative analysis of experiments performed in triplicate in (C) **D.** Silencing of AR by using shRNA. **E.** Reduced cell viability in txr cells following AR silencing. SF, sensitization factor calculated as the ratio of IC_50_ between control shLuc and shAR treatment. The experiments were performed in triplicate (*p < 0.05, **P < 0.01, ***P < 0.005).

To assess the role of driver genes, we selected AR for functional study since this gene possesses many functions and its dysregulation is associated with cancer. DNA microarray analysis indicated a slight increase of AR transcript in SKOV3/Tx50 and around 1.5-fold increase in SKOV3/Tx600 cells compared to parental cells. Using a more quantitative q-PCR analysis, we observed that AR expression was respectively 40-fold and 165-fold higher in SKOV3/Tx50 and SKOV3/Tx600 cells compared to the level of expression in parental cells (Fig. 4A). Notably, both total and nuclear AR protein levels were increased in txr cells, and the level of this protein correlated with the extent of resistance (Fig. 4B–4C and 3K). Silencing of AR using shRNA reduced AR mRNA expression (Fig. 3A), and sensitized txr cells to taxol-induced apoptosis and cell death (Fig. 3A and Fig. 4D–4E). These results indicate that AR expression and its localization to the nucleus may be associated with txr.

### AR activity positively regulates txr genes

**Figure 5:**
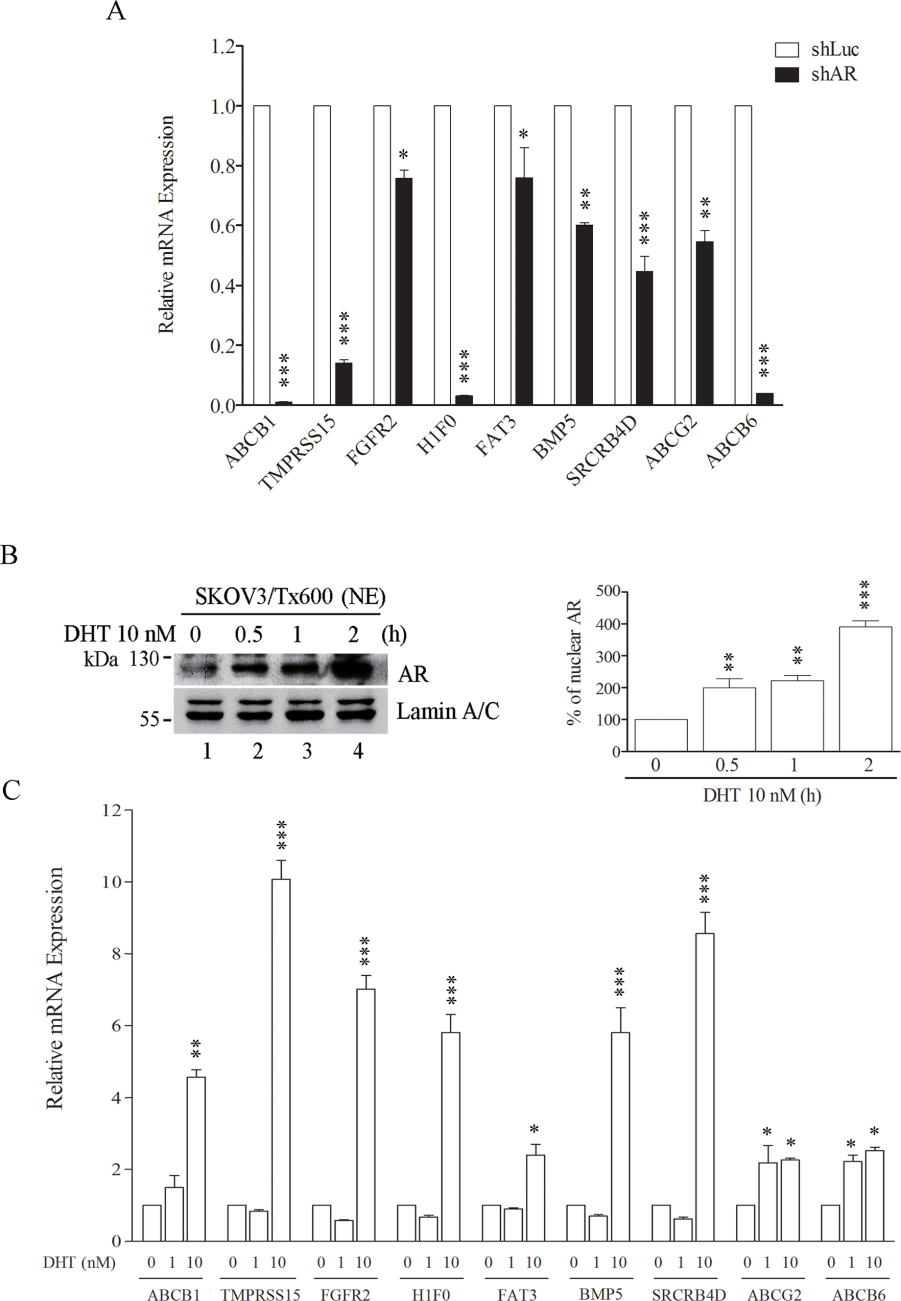
AR activity regulates target txr genes **A.** Downregulation of txr genes following AR silencing. **B.** The nuclear translocation of AR in txr cells increased with the duration of DHT treatment. **C.** Upregulation of txr genes following AR activation using DHT. The experiments were performed in triplicate (*p < 0.05, **P < 0.01, ***P < 0.005).

To assess whether AR induces expression of the potential txr genes, we performed loss-of-function and gain-of-function experiments to monitor the regulation of nine highly upregulated txr genes. All potential txr genes were downregulated in SKOV3/Tx600 cells following silencing of AR (Fig. 5A). In contrast, activation of AR by the agonist DHT (which produced a dose-dependent increase of nuclear AR levels, Fig. 5B) dramatically enhanced the expression of txr genes (Fig. 5C). These results indicate that AR drives the expression of the target txr genes.

### Silencing of AR-target txr genes causes taxol sensitization

**Figure 6:**
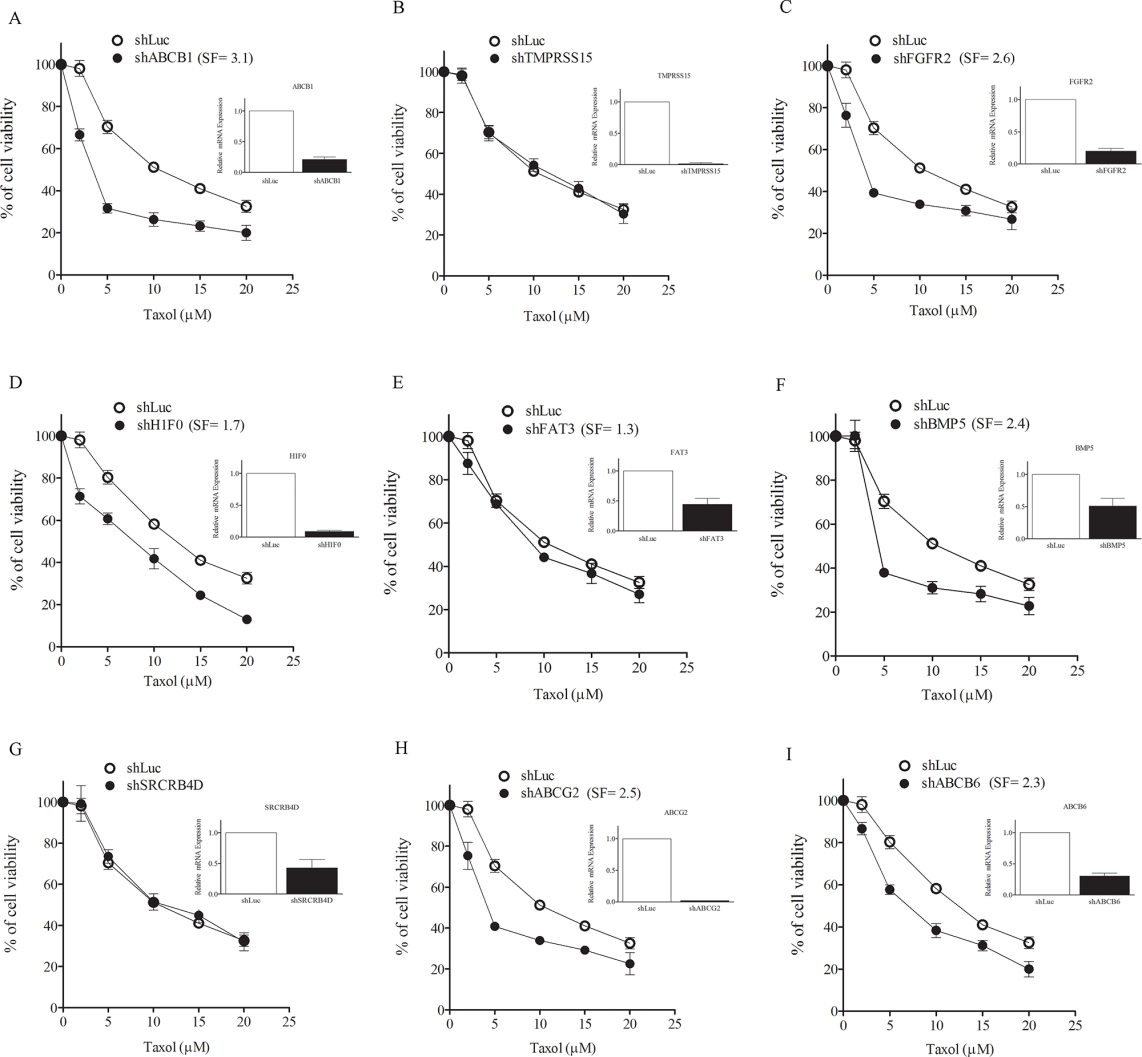
Silencing of txr genes causes taxol sensitization **A–I.** Modulation of cell viability following silencing of txr genes. Each panel shows sensitization of treated cells to taxol with SFs indicated. The results shown were derived from experiments performed in triplicate. Silencing of TMPRSS15 (B) and SRCRB4D (G) showed no impact on cell viability. The inserts shown in each panel indicates gene silencing efficiency as shown by mRNA levels.

To clarify the role of AR-target genes, each potential txr gene was silenced using shRNA. Silencing of txr genes sensitized SKOV3/Tx600 cells cells to taxol to a high level (ABCB1, FGFR2, BMP5, ABCG2, ABCB6), moderate level (H1F0), or low level (FAT3), whereas no sensitization was noted for TMPRSS15 and SRCRB4D (Fig. 6). These results indicate that the AR-target genes tested (7/9 or 78%) are also involved in txr. Furthermore, drug sensitization produced by silencing of these txr genes could also be found in the ovarian carcinoma cell lines MDAH-2774 and TOV21G, as seen for example when FGFR2 was silenced (SF_50_=1.3 and 2.2, respectively) (Fig. S1).

### Identification of AKT pathway as a target of taxol in regulating AR activity and cell sensitivity

**Figure 7:**
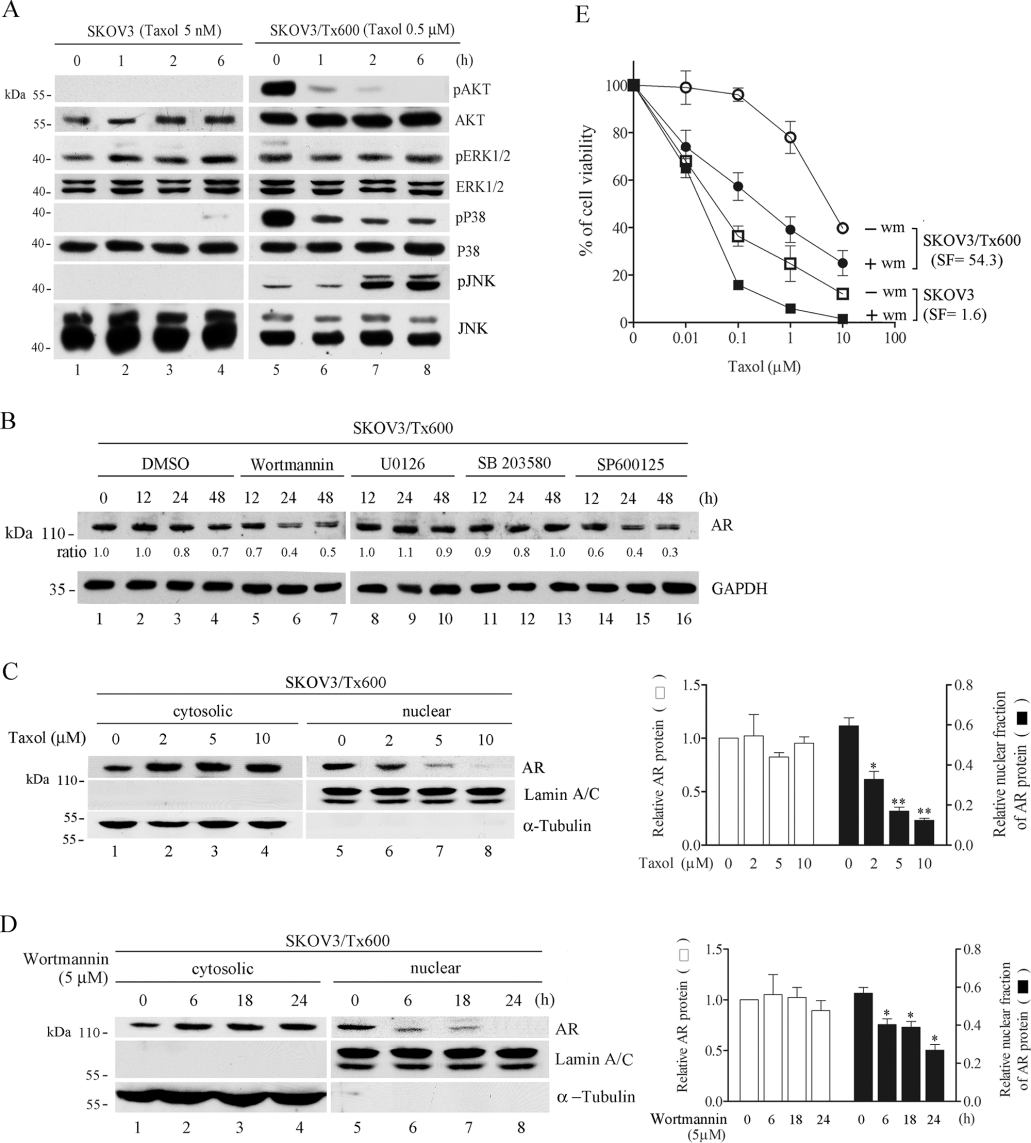
AKT pathway as a target of taxol in regulating AR activity **A.** Enhanced kinase activity in txr cells is sharply inhibited by taxol. AKT and p38 activation was greatly enhanced in txr cells but the level of these proteins was inhibited in a time-dependent manner by taxol. **B.** Dramatic inhibition of AR protein level by inhibitor of AKT and JNK. Wortmannin, AKT inhibitor; U0126, ERK inhibitor; SB203580, p38 inhibitor; SP600125, JNK inhibitor. **C.** Inhibition of pART and AR by taxol. The same concentrations of taxol as in Figure 1A was used here. **D.** Dose-dependent inhibition of nuclear translocation by taxol. **E.** Time-dependent inhibition of nuclear AR translocation by the AKT inhibitor. Lamin A/C and a-tubulin were used to confirm cytosolic and nuclear fractions. (F) Cell viability in response to taxol in the presense or absence of Wortmannin (Wm), an AKT inhibitor. The sensitization factors (SF) of IC_50_ for each gene are indicated. Statistical analysis of three experiments, including total AR and nuclear AR, is shown in the right panel.

To identify the pathways mediating the effects of AR activation, we treated cells with taxol to induce activation of the major kinases. Assuming that kinase activation is required for the effects of AR activation, inhibition of kinase activity should cause a reduction of AR expression level or activity. Both parental cells and SKOV3/Tx600 cells were exposed to equitoxic concentration of taxol. Activation of AKT and p38 in the txr cells was rapidly inhibited by taxol (Fig. 7A, lanes 5–8). While ERK1/2 activation minimally increased in txr cells and was also inhibited by taxol, JNK activation in txr cells was induced by taxol. In contrast, all kinase activities were minimally or not induced by taxol in parental cells (see Fig. 7A, lanes 1–4). Treatment of SKOV3/Tx600 cells with inhibitor of these kinases indicated that only AKT and JNK inhibition (Wortmannin and SP600125, respectively) downregulated AR expression (Fig. 7B). These results suggest that the AKT pathway is likely to represent a target of taxol in regulating AR expression. Supporting this possibility, we observed that treatment with the same taxol concentration used in Fig. 1A produced similar changes in pAKT and AR protein levels (Fig. 7C). The nuclear AR fraction was also examined in txr cells exposed to taxol or Wortmannin. Nuclear AR levels were dramatically inhibited by taxol in a dose-dependent manner (Fig. 7D). While cytosolic AR levels remained unchanged following treatment with various concentrations of taxol, nuclear AR levels were reduced in a dose-dependent manner by taxol (Fig. 7D). Similarly, nuclear AR levels were reduced in a time-dependent manner by Wortmannin (Fig. 7E). To verify the biological significance of the Akt pathway in mediating taxol sensitivity, we treated SKOV3 and SKOV3/Tx600 cells with taxol in the presence or absence of Wortmannin. The sensitization factor obtained was 1.6 and 54.3 for SKOV3 and SKOV3/Tx600 cells, respectively (Fig. 7F), supporting the notion that this pathway is involved in AR-mediated txr.

## DISCUSSION

In this study, we identified drivers of a panel of genes involved in txr. Although these driver genes were not considerably overexpressed, they displayed significant inhibition of taxol sensitivity in our study model. In addition to the driver genes identified, AR, as an example, was shown to activate 13 target txr genes which are highly upregulated in txr cells. Functional studies using 9 of these genes confirmed the biological relevance of the txr genes identified in regulating response to taxol. Silencing a single of these genes (i.e., AR) was sufficient to sensitize txr cells to taxol at levels comparable to the sensitization effect produced by silencing the ABCB1 gene, the typical marker gene whose upregulation is known to be involved in multidrug resistance [4]. The use of the method developed here appears to be critical for the identification of these genes since the driver genes identified were not considerably overexpressed (thus the use of the term cryptic to describe them) in txr cells based on microarray data. This method may thus be useful to identify further driver genes involved in chemoresistance.

Among 2,677 genes that were differentially expressed in txr cells, we found 1,194 upregulated genes in SKOV3 txr cells. Using MetaCore analysis, we identified AR as a leading driver of gene expression for susceptibility genes associated with the txr phenotype, including membrane proteins (ABCB1, ABCB6, ABCG2, FGFR2, TMPRSS15) and chromatin protein (H1F0). Several other transcription factors (ER, c-Myc, AP-1, STAT3, PPAR-gamma) are also found each to be important for upregulation of a group of txr candidate genes. AR recruitment is significantly altered during disease progression and this can arise through changes in the expression of other transcription factors and chromatin modifiers. Proteins that regulate AR activity and the AR-regulated transcriptome have been identified, including transcription factors (c-Myc, STATs, NFκB, ETV1, and ERG) and chromatin modifiers (EZH2, bromodomains, and FOXA1; reviewed in ref. [29]). Notably, the AR-driven genes identified earlier by others as being critical for prostate cancer progression appear to overlap to some extent with the AR-related txr genes identified here in ovarian cancer. Short (CAG)n repeat lengths in AR, altered expression and activity of AR co-activators, and/or differential expression of androgen-mediated genes likely also influence cancer biology and clinical outcome in ovarian epithelial cancer cells [30]. Thus, AR expression or its activity through interaction with other factors appears to be critical for ovarian oncogenesis as well as therapy efficacy. Identification of AR as a driver for stimulating drug resistance genes in ovarian cancers, as demonstrated in this study, should improve our understanding of taxol sensitivity and resistance in ovarian cancer.

Upregulation of AR and other transcription factor genes identified were not easily detectable by transcriptome profiling, possibly due to low expression levels in txr cells. The AR transcript, for example, was found to be upregulated 1.5 fold in taxol-resistant cell lines compared to their taxol-sensitive counterparts based on the microarray data. However, using q-PCR, the AR transcript was found to be upregulated more than 160 fold in taxol-resistant cell lines. The overexpression of AR in txr cells was confirmed by measurement of its protein level, an observation which indicated a discrepancy between microarray and protein expression data. This discrepancy may be partly explained by the fact that the activity of transcription factors is usually regulated by post-translational modifications. For example, AR activity is inhibited by phosphorylation at serine residues by the AKT kinase pathway in a cell-type and cell stage-dependent manner [31–35]. This phosphorylation of AR may lead to Mdm2-mediated protein degradation in prostate cancer cells [32]. Nevertheless, the nuclear fraction of AR was significantly reduced following inhibition of the AKT pathway in ovarian cancer cells (Fig. 7D), an observation which may be due to another cellular signal. The discrepancy in the upregulated AR transcript level in txr cells as determined by microarray and qPCR also indicates possible pitfalls of the microarray analysis. Nevertheless, a combined analysis of microarray data and MetaCore as performed here suggests that important txr genes like AR may be overlooked by single biochemical assays.

We found that the transcription factors c-Myc, AP-1, and STAT3 highly upregulated their target txr genes in ovarian cancer cells. Crosstalk between cellular pathways may also explain the potent AR activity in upregulation of txr genes. The evidence of reciprocal regulation of c-Myc and AR expression, co-expression in castration-resistant prostate cancer, and ligand-independent AR activation by c-Myc [36–38] may explain the genomic instability or metabolic changes observed in prostate cancer. Furthermore, enhanced AR expression and/or copy number amplification by increased STAT5 activation may promote lipid and androgen biosynthesis, as well as dysregulated cell cycle and DNA synthesis in prostate cancer cells [36, 39–42]. We also found that AR binds the Akt-dependent FKBP5 immunophillin, enhancing its transactivation activity, an observation which suggests that this protein may represent a key marker of txr in ovarian cancer cells [28]. Accordingly, enhanced activity of AR and the transcription factors through crosstalk of their pathways may cause genomic instability or metabolic changes via overexpression of target txr genes, resulting in drug resistance phenotype in ovarian cancer cells.

The fact that AR targets 13 prominent txr genes was easily revealed by transcriptome profiling due to high levels of overexpression. In our cell model, the level of upregulation of these txr genes was more or less unstable following repeated cell culture passages (data of this study: GEO database GSE58840; GSE60335 [28]). Some marker genes like ABCB1 which was overexpressed over 100 fold showed reduced levels of overexpression in txr cells after prolonged culture, suggesting that the upregulation of some of these genes may be reversible and epigenetically regulated [43]. Unlike overexpression of ABCB1 in other cell systems which occurs through DNA amplification [4, 5], we noted that overexpression of ABCB1 among these txr genes in SKOV3/Tx600 cells was controlled at the transcriptional level. This observation may be explained by the variable AR activity during cell passage as observed in prostate cancer cells [33]. AR-mediated gene regulation of potential txr genes may be less important in advanced cancer cells in which stable ABCB1 amplification is dominantly responsible for the multidrug resistance phenotype. Transcriptional regulation of potential txr genes by certain key drivers such as AR appears to be important for the initiation and maintenance of txr.

Further studies of txr gene products and other interactors, including chromatin modifiers and co-regulators, are needed to understand the tuning of AR function in txr development. The cellular model developed here will be useful to investigate the molecular events underlying the development of the txr phenotype. A DNA microarray analysis revealed over 120 AR-upregulated genes in OVCAR3 ovarian cancer cells, with the majority being related to transcription, proliferation, and G-protein signaling [44]. Since the dataset obtained in the latter study was not deposited in a public database, our transcriptome profiling of txr genes in SKOV3 cells is probably the first available dataset which can serve as a platform for future studies. More importantly, the strategy presented here provides the possibility to identify and study the genes responsible for txr in ovarian cancer.

## SUPPLEMENTARY FIGURE



## Abbreviations

AR: androgen receptor
DHT: dihydrotestosterone
DMSO: dimethyl sulfoxide
FBS: fetal bovine serum
GAPDH: glyceraldehyde-3-phosphate dehydrogenase
MDR: multiple drug resistance
MTT: 3-(4,5-dimethylthiazol-2-yl)-2,5-diphenyltetrazolium bromide
PBS: phosphate-buffered saline
PCR: polymerase chain reaction
PI3K: phosphatidylinositol 3-kinase
PVDF: polyvinylidene fluoride
q-PCR: quantitative real-time polymerase chain reaction
SDS-PAGE: sodium dodecyl sulfate-polyacrylamide gel electrophoresis
shRNA: short-hairpin RNA
