# Supplementary material for: Integrative transcriptomics-based identification of cryptic drivers of taxol-resistance genes in ovarian carcinoma cells: Analysis of the androgen receptor
Source: Oncotarget. 2015 Aug 11;6(29):27065–82. doi: 10.18632/oncotarget.4824 (PMC4694974; doi:10.18632/oncotarget.4824)
Supplement: Supplementary file 1 [file oncotarget-06-27065-s001.pdf]

SUPPLEMENTARY FIGURE

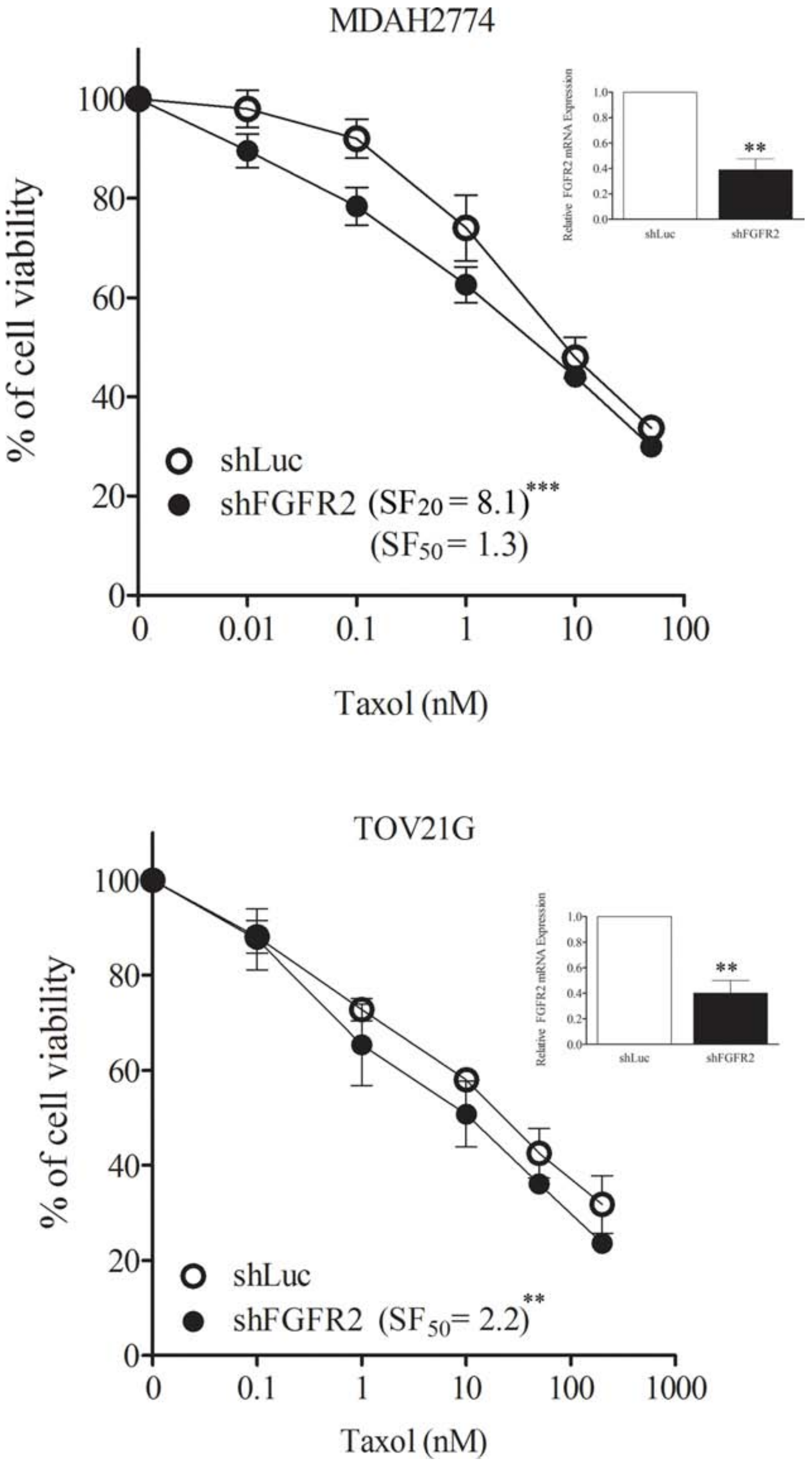

Supplementary Figure S1: Silencing of FGFR2 gene causes taxol sensitization in MADH-2774 and TOV21G ovarian carcinoma cells. Each panel shows sensitization of treated cells to taxol with the SFs indicated. Results shown were derived from experiments performed in triplicate. The inserts shown in each panel indicates gene silencing efficiency as shown by mRNA levels.
